# A Low Serum Calcium‐to‐Potassium Ratio Predicts Postpartum Venous Thromboembolism Risk: Mediation by Hemoglobin and D‐Dimer in a Multicenter Cohort

**DOI:** 10.1002/mco2.70843

**Published:** 2026-06-25

**Authors:** Qian Li, Guofu Zhang, Xiating Li, Huafang Wang, Jun Deng, Zhipeng Cheng, Fengjuan Fan, Shi Chen, De Li, Liang V. Tang, Yu Hu

**Affiliations:** ^1^ Institute of Haematology Union Hospital Tongji Medical College Huazhong University of Science and Technology Wuhan Hubei China; ^2^ Key Laboratory of Biological Targeted Therapy (Huazhong University of Science and Technology) Ministry of Education Wuhan Hubei China; ^3^ School of Public Health Xinxiang Medical University Xinxiang Henan China; ^4^ Diagnostic Science Centre Alberta Precision Laboratories Calgary Alberta Canada; ^5^ Department of Biobank Union Hospital Tongji Medical College Huazhong University of Science and Technology Wuhan Hubei China; ^6^ Collaborative Innovation Center of Hematology Huazhong University of Science and Technology Wuhan Hubei China

**Keywords:** electrolyte, hemoglobin, postpartum, pregnant women, venous thromboembolism

## Abstract

Postpartum venous thromboembolism (VTE) is a serious complication, and electrolytes may play a role in coagulation homeostasis, but their associations with postpartum VTE remain unclear. A total of 25,792 pregnant women from Chinese multicenter cohort between January 2017 and December 2024 were included, and the occurrences of postpartum VTE within 6 weeks after delivery were followed. Here, we show that dysregulated perinatal electrolyte profiles and a reduced serum calcium (Ca) /potassium (K) ratio are novel and independent risk factors for postpartum VTE, with hemoglobin and D‐dimer levels mediating such associations. Restricted cubic spline analyses showed that low serum Ca (overall association *p* = 0.032) and high serum K (overall association *p* = 0.036) were associated with increased postpartum VTE risk. Serum magnesium exhibited a nonlinear inverse U‐shaped association with VTE risk (nonlinear *p* = 0.021). Low serum Ca/K ratio was linked to elevated VTE risk (overall association *p* = 0.004), with a nonlinear relationship (nonlinear *p* = 0.042) and an inflection point at 0.55. Notably, a serum Ca/K ratio below 0.55 is a promising new biomarker for predicting the risk of postpartum VTE. Mediation analyses suggested that the protective effect of a higher Ca/K ratio might be partially explained by its association with higher hemoglobin levels (34.7% mediation, *p* = 0.036) and lower D‐dimer levels (4.6% mediation, *p* = 0.018). This study suggests that strategies aimed at optimizing perinatal electrolyte balance and the monitoring and management of anemia may help reduce the burden of postpartum VTE.

## Graphical abstract

In a large multicenter cohort, a low serum calcium‐to‐potassium (Ca/K) ratio (< 0.55) in the third trimester was a novel risk factor for postpartum venous thromboembolism (VTE). This association was partly explained by concomitant variations in hemoglobin and D‐dimer levels, pointing to integrated management of electrolytes and anemia for postpartum VTE prevention.

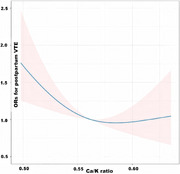

## 1 Introduction

Venous thromboembolism (VTE), which includes deep vein thrombosis (DVT) and pulmonary embolism (PE), is a serious complication that threatens the safety of mother and child. The pooled incidence of perinatal VTE is about 1.4‰ (1.0‰–1.8‰), which is four to five times that of ordinary women of childbearing age [1, 2, 3]. In China, despite the lack of national large‐scale epidemiological survey data, meta‐analyses of 53 studies had shown that the incidence of maternal VTE was 1.3‰ (95% CI, 1.1‰–1.6‰) [4, 5]. At present, it has been clarified that advanced pregnancy (≥ 35 years old), preconception obesity (body mass index ≥ 30), multiple pregnancies, oral contraceptives, non‐delivery‐related hospital admissions, and so on are high‐risk factors for perinatal VTE [2, 5, 6]. To better translate these risk factors into precise prevention strategies in diverse clinical settings, conducting large‐scale real‐world studies is essential [7].

Electrolyte balance is a key link in maintaining maternal and fetal health, and the electrolytes routinely tested in clinical practice include calcium (Ca), magnesium (Mg), sodium (Na), potassium (K), phosphorus (P), and chlorine (Cl). Ca deficiency in pregnant women may lead to long‐term bedridden after fractures due to osteoporosis [8, 9, 10], which indirectly increases the risk of VTE. Mg exerts antithrombotic effects by inhibiting platelet aggregation, promoting the release of prostacyclin (PGI_2_), and reducing vascular endothelial damage [11, 12, 13].

Too high or too low Na intake may increase the risk of cardiovascular disease [14]. Hyperkalemia may increase the risk of thrombosis by inhibiting myocardial function [15]. Hyperphosphatemia in patients with chronic kidney disease is associated with an increased risk of cardiovascular events, including thrombosis, suggesting that P may indirectly affect perinatal thrombosis through vascular calcification [16, 17, 18]. Cl indirectly affects coagulation function by maintaining acid‐base balance: acidosis may enhance clotting factor activity, and alkalosis may inhibit platelet function [19].

Three previous studies have investigated the association of single electrolyte with the risk of blood clots, two of which focused on Ca [20, 21] and one on Mg [22]. Specifically, one randomized controlled trial (RCT) showed that participants who supplemented with Ca and vitamin D had a significantly lower risk of idiopathic VTE compared with the placebo group, but there was no statistical difference in the overall risk of VTE between the two groups [21]. The remaining two cohort studies of Ca [20], Mg [22], and VTE did not find a significant association between Ca or Mg levels and VTE risk. To date, no study has investigated the relationship between six serum electrolytes and the risk of VTE, and no relevant research has been conducted specifically in pregnant populations.

Therefore, in this large, multicenter cohort study, we aimed to systematically evaluate the associations between six serum electrolytes and postpartum VTE risk. We sought to identify the most critical electrolyte markers, establish their risk thresholds, and explore their combined effects. Our findings are expected to uncover novel, accessible biomarkers for VTE risk stratification and provide mechanistic insights to inform future precise prevention strategies.

## 2 Results

### 2.1 Characteristics of the Study Population

In this study, 32,241 pregnant women gave live birth in the fixed research hospitals, 6406 participants without electrolyte indicator measurement in the third trimester were excluded, and 43 pregnant women with a history of VTE before inclusion were excluded, and finally 25,792 pregnant women were included in this study (Figure S1). A total of 25,792 pregnant women were included in this study, with an incidence of postpartum VTE of 4.7 per 1000 (120/25,792). Compared with the non‐VTE group, women in the VTE group were older, had a higher proportion of multiparity, were shorter in height, had a higher cesarean section rate, and showed lower levels of Hb and APTT, as well as higher D‐dimer levels (Table 1). Baseline characteristics were comparable between included and excluded participants (Table S1), and were also similar between participants enrolled from 2017 to 2024 and those enrolled from 2019 to 2020 (after excluding the latter, Table S2).

**TABLE 1:** Characteristics and laboratory findings of the participants of non‐VTE and VTE (*n* = 25,792).

	Total (*N* = 25792)	Non‐VTE (*n* = 25,672)	VTE (*n* = 120)	*p* value
**Characteristics**				
Maternal age (years)	31.0 (28.0, 34.0)	31.0 (28.0, 34.0)	33.0 (30.0, 35.0)	< 0.001^a^
Ethnic group (Han)	25,622 (99.3)	25,503 (99.3)	119 (99.2)	0.549
Multiple pregnancy (%)	1094 (4.2)	1087 (4.2)	7 (5.8)	0.359
Primipara (%)	16807 (65.2)	16744 (65.2)	63 (52.5)	0.005^a^
IVF pregnancy (%)	2035 (7.9)	2024 (7.9)	11 (9.2)	0.608
Height at enrollment (cm)	161.0 (158.0, 165.0)	161.0 (158.0, 165.0)	160.0 (158.0, 163.0)	0.001^a^
Weight at enrollment (kg)	69.0 (63.0, 75.0)	69.0 (63.0, 75.0)	69.0 (63.6, 73.4)	0.650
Habit of smoking (%)	38 (0.1)	37 (0.1)	1 (0.8)	0.163
Habit of drinking (%)	24 (0.1)	23 (0.1)	1 (0.8)	0.106
GDM (%)	5879 (22.8)	5844 (22.8)	35 (29.2)	0.099
Preeclampsia (%)	1193 (4.6)	1185 (4.6)	8 (6.7)	0.272
Delivery mode (cesarean, %)	18,292 (70.9)	1817 (70.8)	117 (97.5)	< 0.001^a^
Postpartum hemorrhage	545 (2.1)	542 (2.1)	3 (2.5)	0.743
Preterm (%)	2911 (11.3)	2894 (11.3)	17 (14.2)	0.311
Chronic kidney disease	88 (0.3)	88 (0.3)	0 (0.0)	0.521
Autoimmune diseases	161 (0.6)	160 (0.6)	1 (0.8)	0.771
**Laboratory findings**				
Hb (g/L)	119.0 (110.0, 127.0)	119.0 (110.0, 127.0)	112.0 (103.0, 122.75)	< 0.001^a^
APTT (s)	32.0 (30.6, 33.7)	32.0 (30.6, 33.7)	31.5 (29.7, 33.8)	0.048^a^
PT (s)	12.2 (11.8, 12.6)	12.2 (11.8, 12.6)	12.2 (11.6, 12.7)	0.700
TT (s)	16.5 (15.7, 17.4)	16.5 (15.7, 17.4)	16.4 (15.6, 17.0)	0.108
Fib (g/L)	4.71 (4.22, 5.25)	4.72 (4.22, 5.25)	4.64 (4.16, 5.17)	0.305
D‐dimer (g/L)	1.43 (1.04, 2.01)	1.43 (1.03, 2.01)	1.62 (1.20, 2.24)	0.005^a^
CRP	2.55 (1.24, 5.73)	2.55 (1.24, 5.72)	3.60 (1.60, 7.73)	0.062

Abbreviations: APTT, activated partial thromboplastin time; BMI, body mass index; CRP, C‐reactive protein; Fib, fibrinogen; GDM, gestational diabetes mellitus; Hb, hemoglobin; IVF, in vitro fertilization; PT, prothrombin time; TT, thrombin time; VTE, venous thromboembolism.

^a^ *p* < 0.05.

### 2.2 Correlation of the Six Electrolytes

Spearman correlation analysis was performed on six electrolytes, and it was found that except for serum K and Cl, correlations were not significant, there were mild significant positive or negative correlations between the other electrolytes, and the correlation coefficients ranged from −0.195 to 0.260 (Figure S2).

### 2.3 Restricted Cubic Spline (RCS) Analyses of Six Electrolytes and Postpartum VTE

**FIGURE 1:**
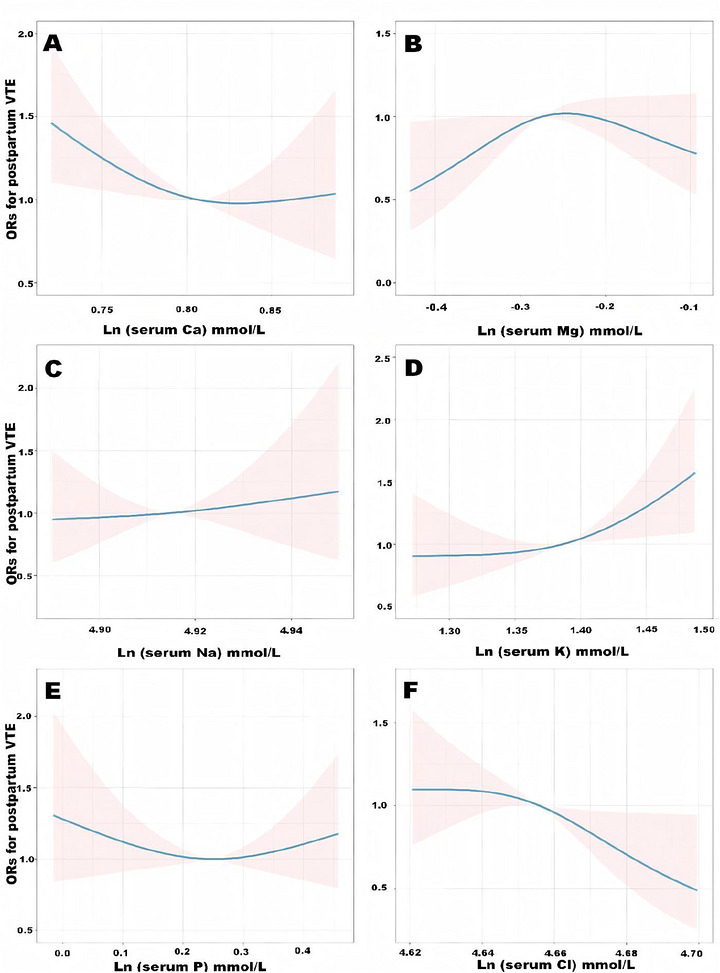
Adjusted restricted cubic spline (RCS) analyses of associations of six ln‐transformed serum electrolytes and the risk of postpartum VTE (*n* = 25,792). The RCS for the association between ln‐transformed serum Ca and the risk of postpartum VTE. (B) The RCS for the association between ln‐transformed serum Mg and the risk of postpartum VTE. (C) The RCS for the association between ln‐transformed serum Na and the risk of postpartum VTE. (D) The RCS for the association between ln‐transformed serum K and the risk of postpartum VTE. (E) The RCS for the association between ln‐transformed serum P and the risk of postpartum VTE. (F) The RCS for the association between ln‐transformed serum Cl and the risk of postpartum VTE. Ca: calcium; Cl: chlorine; K: potassium; Mg: magnesium; Na: sodium; OR: odds ratio; P: phosphorus; VTE: venous thromboembolism. Models of A, B, C, D, E and F adjusted for maternal age, multiple pregnancy, primipara, in vitro fertilization pregnancy, ethnic group, BMI at enrollment, habit of smoking, habit of drinking, gestational diabetes mellitus, preeclampsia, preterm, delivery mode, postpartum hemorrhage, chronic kidney disease, autoimmune diseases, and other five serum electrolytes.

RCS analyses of the associations of natural log‐transformed serum Ca, Mg, Na, K, P, Cl, and postpartum VTE risk showed that low serum Ca (*p* of overall association = 0.032) and high serum K (*p* of overall association = 0.036) were associated with an increased risk of postpartum VTE (Figure 1A,D). Serum Mg exhibited a nonlinear (inverse U‐shaped) association with postpartum VTE risk (*p* of nonlinear association = 0.021), with an inflection point at 0.76 mmol/L (Figure 1B). No significant associations were observed for the remaining three electrolytes and postpartum VTE risk (all *p* > 0.05, Figure 1C,E,F).

### 2.4 Weighted Quantile Sum (WQS) Analyses of Six Electrolytes and Postpartum VTE

**FIGURE 2:**
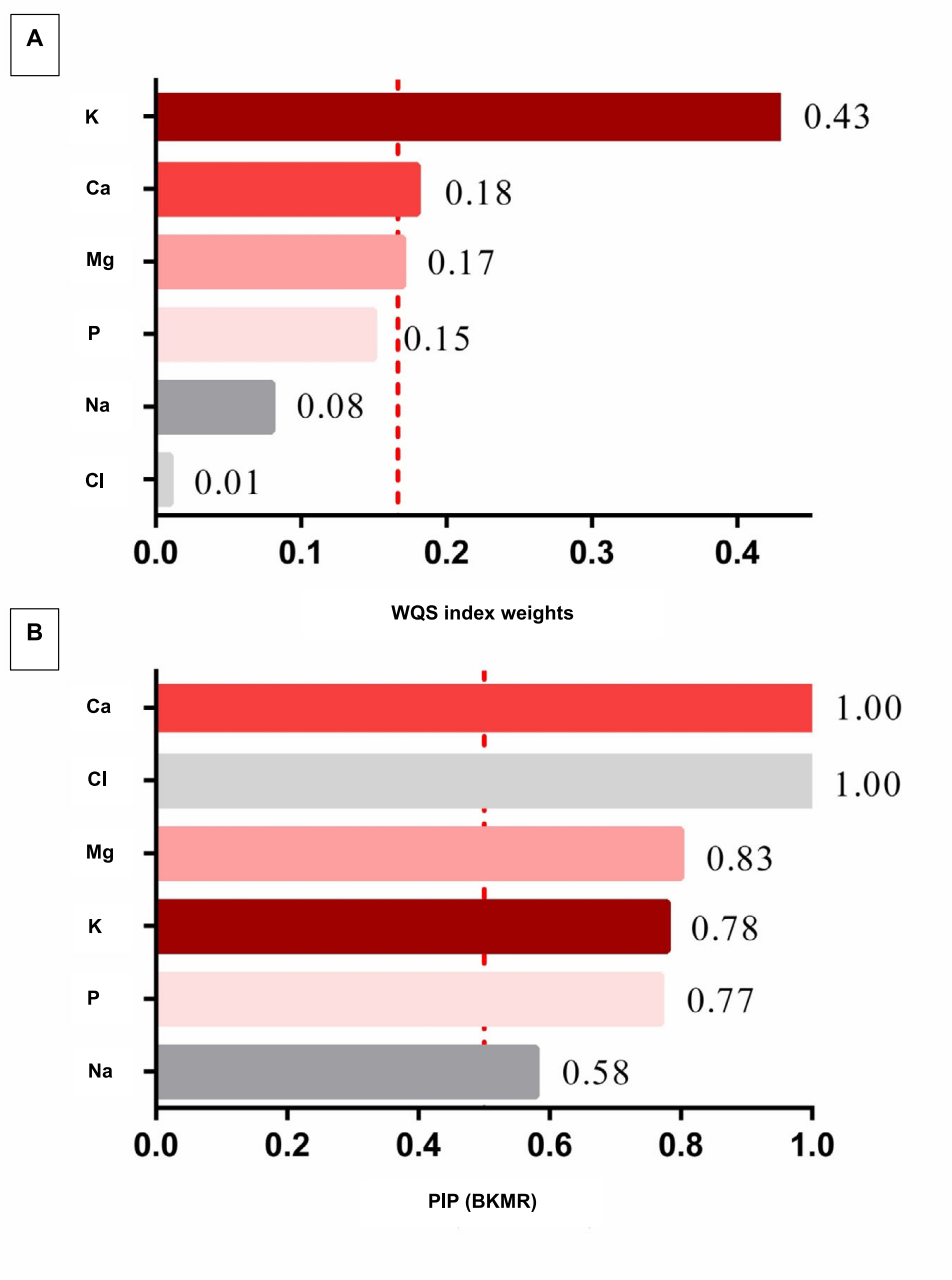
Weighted Quantile Sum (WQS, A) and Bayesian Kernel Machine Regression (BKMR, B) analyses of associations between six serum electrolytes and postpartum VTE. Ca: calcium; Cl: chlorine; K: potassium; Mg: magnesium; Na: sodium; OR: odds ratio; P: phosphorus; VTE: venous thromboembolism. Adjusted for maternal age, multiple pregnancy, primipara, in vitro fertilization pregnancy, ethnic group, BMI at enrollment, habit of smoking, habit of drinking, gestational diabetes mellitus, preeclampsia, preterm, delivery mode, postpartum hemorrhage, chronic kidney disease, and autoimmune diseases.

WQS analyses found no significant association between mixed exposure to the six electrolytes and postpartum VTE risk: the unadjusted model showed an odds ratio (OR) of 1.03 (95% confidence interval [CI]: 0.72, 1.46), and the adjusted model showed an OR of 1.06 (95% CI: 0.74, 1.52). In the WQS model, K contributed the most to the weight (0.43), followed by Ca (0.18), Mg (0.17), and P (0.15); Na (0.08) and Cl (0.01) had smaller contributions (Figure 2A).

### 2.5 Bayesian Kernel Machine Regression (BKMR) Analyses of Six Electrolytes and Postpartum VTE

**FIGURE 3:**
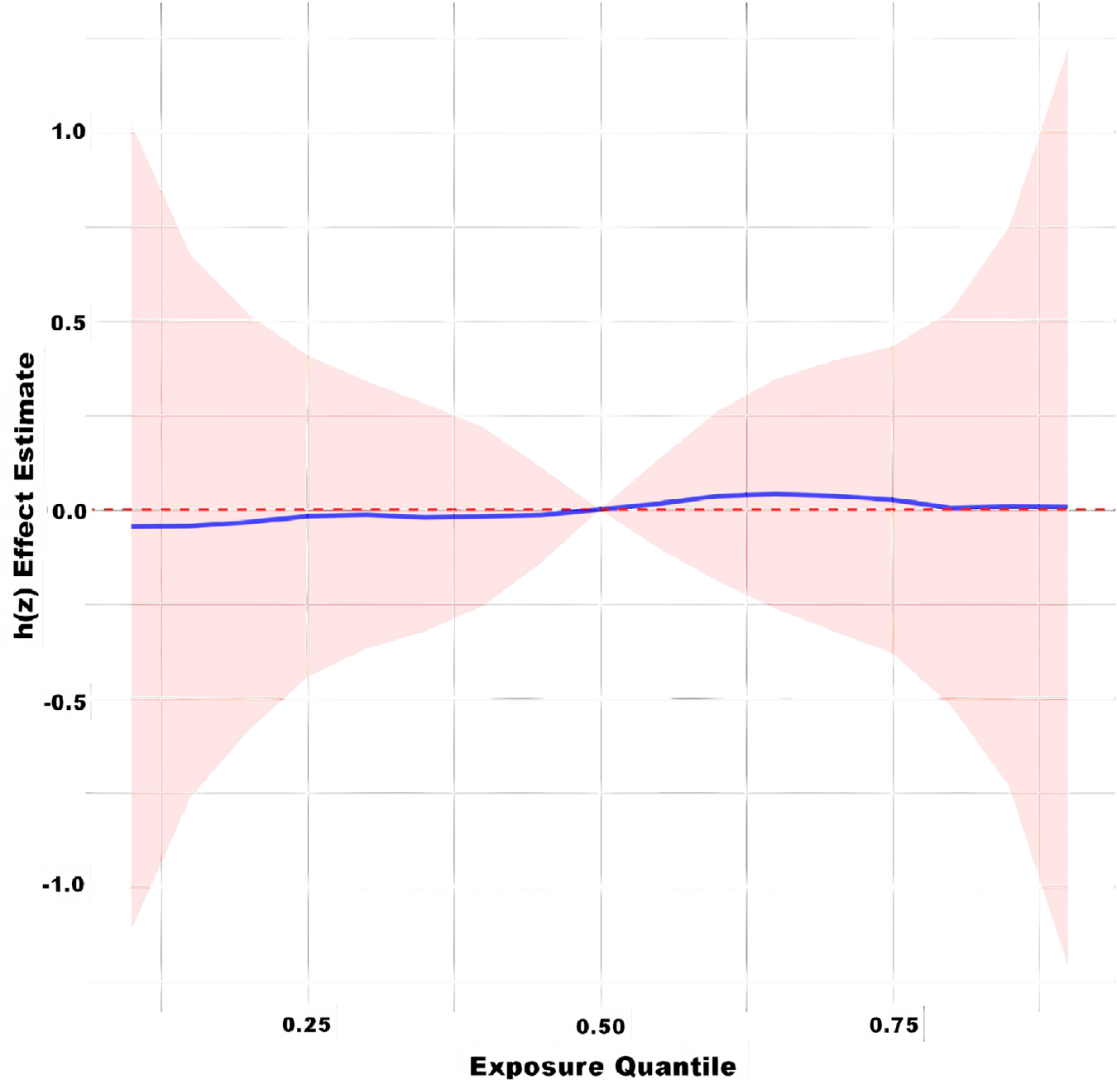
Joint effect (95% credible intervals, CIs) of the six serum electrolytes on postpartum VTE by using Bayesian kernel machine regression (BKMR) model. The figure plots the estimated change in a latent continuous outcome (continuous marker of the binary postpartum VTE when all the electrolytes at fixed percentiles were compared to all the electrolytes at their 50th percentile). Ca: calcium; CI: confidence interval; Cl: chlorine; K: potassium; Mg: magnesium; Na: sodium; OR: odds ratio; P: phosphorus; VTE: venous thromboembolism. Adjusted for maternal age, multiple pregnancy, primipara, in vitro fertilization pregnancy, ethnic group, BMI at enrollment, habit of smoking, habit of drinking, gestational diabetes mellitus, preeclampsia, preterm, delivery mode, postpartum hemorrhage, chronic kidney disease, and autoimmune diseases.

**FIGURE 4:**
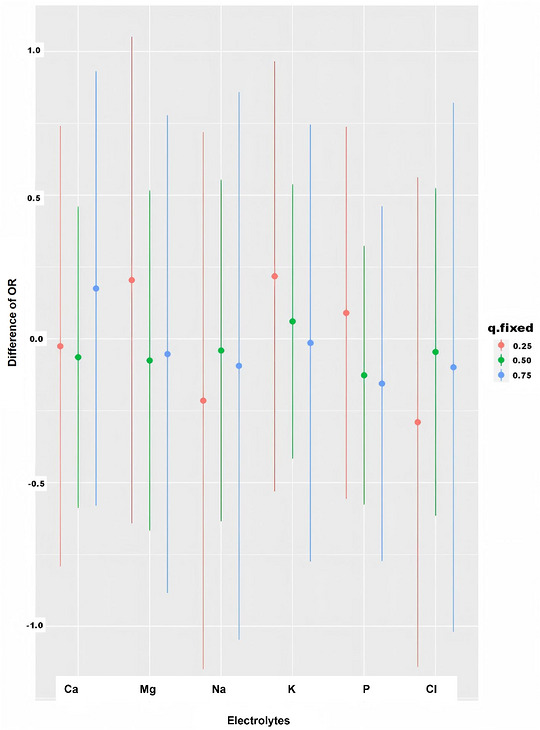
Conditional single‐electrolyte associations (estimates and 95% CI) with postpartum VTE risk. CI: confidence interval; VTE: venous thromboembolism. This plot compares a latent continuous outcome when a single electrolyte is at the 75th versus 25th percentile, when all the other exposures are fixed at either the 25th, 50th, or 75th percentile.

BKMR analyses of the effect of mixed exposure to the six electrolytes on postpartum VTE risk also found no significant association (Figure 3). In the BKMR model, the posterior inclusion probabilities (PIP) were highest for Ca (PIP = 1) and Cl (PIP = 1), followed by Mg (PIP = 0.83), K (PIP = 0.78), and P (PIP = 0.77); Na had the lowest PIP (0.58) (Figure 2B). The effect of single electrolyte (75th vs. 25th percentile) on the risk of postpartum VTE was nonsignificant when the concentrations of the other five electrolytes were fixed at 25th, 50th, and 75th percentiles (Figure 4).

### 2.6 RCS Analyses of Ca/K Ratio and Postpartum VTE

**FIGURE 5:**
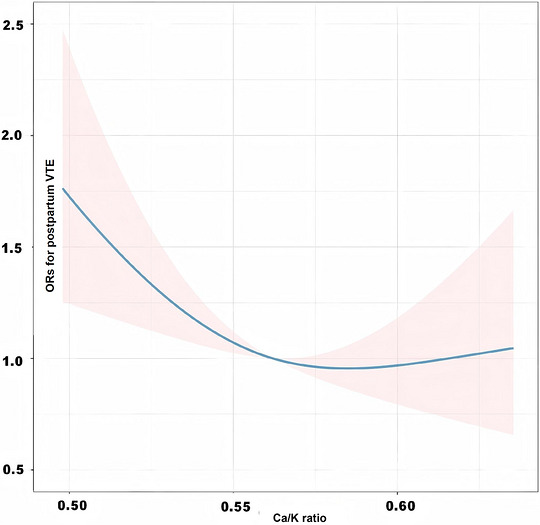
Restricted Cubic Spline (RCS) analyses of associations between Ca/K ratio and postpartum VTE. Ca: calcium; K: potassium; OR: odds ratio; VTE: venous thromboembolism. Models adjusted for maternal age, multiple pregnancy, primipara, in vitro fertilization pregnancy, ethnic group, BMI at enrollment, habit of smoking, habit of drinking, gestational diabetes mellitus, preeclampsia, preterm, delivery mode, postpartum hemorrhage, chronic kidney disease, autoimmune diseases, serum magnesium, serum sodium, serum phosphorus, and serum chlorine.

RCS analysis of the Ca/K ratio showed that a low serum Ca/K ratio was associated with an increased risk of postpartum VTE (*p* of overall association = 0.004), with a nonlinear association (nonlinear association = 0.042) and an inflection point at 0.553 (95%CI, 0.521, 0.585) (Figure 5). Compared with the group with a Ca/K ratio ≥ 0.553, the group with a ratio < 0.553 had an increased risk of postpartum VTE (OR, 1.57 [1.08, 2.28], Table 2). When the cut‐off value was replaced with 0.55, the low Ca/K ratio group still showed a higher risk of postpartum VTE (OR, 1.57 [1.08, 2.28], Table 2). Stratified analysis is shown in Table 3. The association between a low Ca/K ratio and increased postpartum VTE risk remained significant after excluding participants with chronic kidney disease, autoimmune diseases, and those enrolled from 2019 to 2020 (Table S3). To address potential confounding by serum albumin, we performed a sensitivity analysis using the albumin‐corrected Ca/K ratio. The RCS analysis of the corrected ratio continued to show a significant inverse overall association with postpartum VTE risk (overall *p* = 0.039), confirming the primary finding (Figure S3). While the nonlinear component was attenuated and no longer statistically significant in this model, the dose–response curve consistently indicated higher VTE risk at lower values of the corrected ratio.

**TABLE 2:** Logistic regression of calcium‐potassium (Ca/K) ratio and postpartum VTE risk (*n* = 25,792).

Categories	Unadjusted OR (95% CI)	Adjusted OR (95% CI)
**Ca/K ratio**		
< 0.553	1.60 (1.12, 2.29)^*^	1.57 (1.08, 2.28)^*^
≥ 0.553	Ref.	Ref.
**Ca/K ratio**		
< 0.55	1.87 (1.55, 2.45)^*^	1.57 (1.08, 2.28)^*^
≥ 0.55	Ref.	Ref.

*Note*: Adjusted for maternal age, multiple pregnancy, primipara, in vitro fertilization pregnancy, ethnic group, BMI at enrollment, habit of smoking, habit of drinking, gestational diabetes mellitus, preeclampsia, preterm, delivery mode, postpartum hemorrhage, chronic kidney disease, autoimmune diseases, serum magnesium, serum sodium, serum phosphorus, and serum chlorine.

Abbreviations: Ca, calcium; CI, confidence interval; K, potassium; OR, odds ratio; VTE, venous thromboembolism.

* *p* < 0.05.

**TABLE 3:** Stratified analysis of calcium–potassium (Ca/K) ratio and postpartum VTE risk (*n* = 25,792).

Categories	Ca/K ratio		*p* for interaction
	< 0.55	≥ 0.55	
**Delivery mode** ^a^	OR (95% CI)		0.732
Cesarean section	1.51 (1.05, 2.18)^*^	Ref.	
Vaginal delivery	0.99 (0.05, 10.34)	Ref.	
**Primipara** ^b^	OR (95% CI)		0.516
Yes	1.80 (1.10, 2.97)^*^	Ref.	
No	1.42 (0.83, 2.39)	Ref.	

Abbreviations: Ca, calcium; CI, confidence interval; Cl, chlorine; K, potassium; Mg, magnesium; Na, sodium; OR, odds ratio; P, phosphorus; VTE, venous thromboembolism.

^a^ Adjusted for maternal age, multiple pregnancy, primipara, in vitro fertilization pregnancy, ethnic group, BMI at enrollment, habit of smoking, habit of drinking, gestational diabetes mellitus, preeclampsia, preterm, postpartum hemorrhage, chronic kidney disease, autoimmune diseases, serum magnesium, serum sodium, serum phosphorus, and serum chlorine.

^b^ Adjusted for maternal age, multiple pregnancy, in vitro fertilization pregnancy, ethnic group, BMI at enrollment, habit of smoking, habit of drinking, gestational diabetes mellitus, preeclampsia, preterm, delivery mode, postpartum hemorrhage, chronic kidney disease, autoimmune diseases, serum magnesium, serum sodium, serum phosphorus, and serum chlorine.

* *p* < 0.05.

### 2.7 Mediation Analyses of Ca/K Ratio and Postpartum VTE

Mediation analyses revealed two significant indirect pathways linking a higher Ca/K ratio to a lower risk of postpartum VTE: one through higher Hb levels, accounting for 34.7% of the total effect (*p* = 0.036), and another through lower D‐dimer levels, accounting for 4.6% (*p* = 0.018) (Table S4). No significant mediating roles were observed for CRP, APTT, PT, or FIB in the association between the Ca/K ratio and postpartum VTE risk (Table S4).

### 2.8 Population Attributable Risk Percentage (PAR%) of Ca/K Ratio in Postpartum VTE

Furthermore, elimination of the adverse effects of a low Ca/K ratio could reduce the PAR% of postpartum VTE by 17.7%.

### 2.9 Interactive Analyses Between Chlorine and Magnesium on Postpartum VTE Risk

Analysis of the interaction between Cl and Mg on postpartum VTE risk revealed a significant interaction effect (*p* = 0.040). After categorizing serum Cl and Mg into tertiles, the low‐Cl and medium‐Mg group had an increased risk of postpartum VTE compared with the medium‐Cl and low‐Mg group (OR, 3.19 [1.37, 7.42]) (Figure S4). When participants were divided into the low‐Cl and medium‐Mg group versus other groups, the low‐Cl and medium‐Mg group showed an increased risk of postpartum VTE (OR, 2.11 [1.34, 3.31]). Stratified by GDM, the increased risk of postpartum VTE in the low‐Cl and medium‐Mg group (compared with other groups) was only observed in non‐GDM pregnant women (OR, 2.04 [1.20, 3.48]) (Table S5).

## 3 Discussion

This study demonstrated that pregnant women with a serum Ca/K ratio < 0.55 had 1.57‐fold risk of postpartum VTE compared to those with a ratio ≥ 0.55. The increased postpartum VTE risk associated with a low serum Ca/K ratio was primarily observed in pregnant women who underwent cesarean section and in multiparous women. Mediation analyses revealed that the association between the serum Ca/K ratio and postpartum VTE risk was partially mediated by Hb and D‐dimer levels. Furthermore, elimination of the adverse effects of a low serum Ca/K ratio could reduce the PAR% by 17.7%.

For the first time, this study investigated the associations between serum electrolytes and postpartum VTE risk, with a focus on their individual, combined, and ratio‐based effects, as well as mediating mechanisms. Our findings reveal several key insights that advance understanding of electrolyte homeostasis in perinatal thromboprophylaxis, highlighting electrolyte balance and the monitoring and management of anemia as key, modifiable targets.

Prior to the present study, only three studies had explored the association between electrolytes and VTE risk, with a focus on Ca (two studies) and Mg (one study). Lerstad et al. conducted a cohort study in Norway and found no significant association between serum Ca levels and VTE risk; the hazard ratio (HR) for the highest versus lowest quartile of serum Ca was 0.95 (95% CI: 0.77–1.17), indicating no meaningful difference [20]. In a RCT involving postmenopausal women in the United States, Blondon et al. observed that participants receiving 1000 mg of Ca carbonate plus 400 IU of vitamin D3 daily had a reduced risk of idiopathic VTE compared with the placebo group (relative risk [RR] = 0.62, 95% CI: 0.42–0.92), though no significant difference was noted in overall VTE risk [21]. Additionally, a cohort study by Kunutsor et al. in Finnish men failed to identify a significant association between serum Mg and VTE risk (HR = 1.38, 95% CI: 0.48–3.96 per 1 standard deviation increase in serum Mg) [22]. To our knowledge, the current study is the first to investigate the relationship between electrolyte levels and VTE risk specifically in pregnant women. Furthermore, it is the first to emphasize the importance of electrolyte balance—rather than individual electrolyte levels—in the prevention of postpartum VTE, filling a critical gap in the understanding of perinatal thromboprophylaxis.

Low serum Ca may increase the risk of postpartum VTE by promoting platelet activation and aggregation, enhancing coagulation factor activity, and impairing endothelial barrier function, thereby shifting the hemostatic balance toward a prothrombotic state [23]. High serum K may increase the risk of postpartum VTE by disrupting endothelial cell function, promoting platelet activation, and altering the balance of procoagulant and anticoagulant factors, thereby facilitating the formation of blood clots [24]. The inverse U‐shaped pattern for Mg may be attributed to Mg's dual effects on hemostasis—while moderate concentrations might disrupt the balance between procoagulant and anticoagulant pathways, lower levels could avoid such disruption, and higher levels may exert protective effects by inhibiting excessive platelet aggregation or regulating endothelial function, thereby maintaining coagulation homeostasis [12, 25]. In addition, this may be related to the interaction between Mg and Cl. The current findings highlight the need to move beyond binary “deficiency versus sufficiency” frameworks to consider Mg's dose‐dependent effects on postpartum VTE.

In this study, the Ca/K ratio emerged as a stronger predictor of postpartum VTE risk, with a nonlinear association and a critical threshold (0.55). Our sensitivity analysis using albumin‐corrected Ca provided critical validation. While the specific nonlinear shape observed with total Ca was attenuated, a statistically significant overall inverse association between the corrected Ca/K ratio and VTE risk persisted. This indicates that the core relationship is not driven by albumin‐related factors (e.g., hemodilution, inflammation, or nutritional status). The correction likely removed variance attributable to albumin‐bound Ca, refining the association to more directly reflect the component of Ca homeostasis pertinent to thrombosis risk. Therefore, the central conclusion—that a lower Ca/K ratio is an independent risk factor for postpartum VTE—is strongly supported by both the original and albumin‐corrected analyses.

The absence of a significant association in vaginal delivery or nulliparous groups likely reflects lower baseline postpartum VTE risk in these populations, where other factors (e.g., spontaneous mobilization in vaginal delivery or fewer metabolic perturbations in nulliparas) may override the impact of Ca/K ratio. These findings highlight the importance of tailored risk assessment: monitoring Ca/K ratios could be particularly valuable in cesarean and multiparous cohorts, offering a potential target for personalized thromboprophylaxis to mitigate their elevated postpartum VTE risk. The lack of statistical significance in the vaginal delivery and nulliparous groups may be attributed to two factors: (1) lower baseline VTE risk in these populations; (2) relatively smaller sample sizes in these subgroups, which may have reduced statistical power to detect potential associations. Larger cohort studies are needed in the future.

Mediation analyses identified Hb and D‐dimer as significant mediators in the relationship between Ca/K ratio and postpartum VTE risk. The mediating role of Hb warrants specific interpretation. In the peripartum context, this finding likely indicates that the risk associated with anemia, rather than a benefit of high Hb per se, is partially mitigated by a higher Ca/K ratio. Peripartum anemia can promote venous stasis and endothelial dysfunction, key drivers of thrombosis [26, 27]. Thus, the mediation effect suggests that an adequate Ca/K ratio may help maintain Hb above a threshold that exacerbates anemia‐related thrombotic risk. This study underscore the importance of anemia management in VTE prevention [28, 29]. This work, together with our team's prior studies, collectively underscores a critical research paradigm: identifying and modifying specific, measurable biological derangements is key to mitigating thrombotic and hemorrhagic risks [30, 31].

The population attributable risk percentage (17.7%) associated with low Ca/K ratios suggests that optimizing this ratio could meaningfully reduce postpartum VTE burden. Clinically, monitoring Ca and K levels—either individually or as a ratio—may complement existing risk assessment tools (e.g., Caprini score) to identify high‐risk women [32, 33].

The interaction between Cl and Mg further refined our understanding: low Cl combined with medium Mg was associated with increased postpartum VTE risk, particularly in non‐GDM women. Cl, via its role in acid‐base balance, may modulate coagulation factor activity [19], while Mg's vascular protective effects could be blunted in the context of Cl imbalance. The GDM‐specific stratification suggests that metabolic perturbations in GDM (e.g., insulin resistance) may alter electrolyte‐mediated thrombotic pathways [34].

The divergent key drivers identified by WQS (K) and BKMR (Ca and Cl) analyses reflect their distinct assumptions regarding mixture effects. WQS assumes a unidirectional joint effect, while BKMR non‐parametrically identifies effect modifiers within the network. Importantly, both methods underscore the central roles of Ca and K, reinforcing our core finding that the Ca/K ratio is a critical determinant of postpartum VTE risk. This suggests that the balance between these electrolytes is more physiologically relevant than their absolute concentrations or a simple additive mixture effect. The non‐significant overall association may stem from antagonistic interactions or complex nonlinear relationships within the electrolyte network.

The strengths of this study include the following: First, it enrolled a large sample size of pregnant women from multicenter cohort, which enhances the statistical power and generalizability of the findings. Second, a comprehensive analytical framework was employed, incorporating multiple advanced statistical methods such as RCS analyses to explore dose–response relationships, WQS and BKMR to assess mixed exposure effects, mediation analyses to investigate underlying mechanisms, and interaction analyses to examine combined effects of electrolytes, ensuring robust and multi‐dimensional insights into the associations. Third, the study demonstrated good robustness, as key findings remained significant after excluding specific subgroups. Fourth, it explored potential mediating pathways (via Hb and D‐dimer) and PAR%, providing mechanistic and public health implications. Fifth, stratified analyses (e.g., by GDM) and interaction assessments (e.g., between Cl and Mg) further enriched the understanding of context‐specific effects. Lastly, comparable baseline characteristics between included and excluded participants reduced the risk of selection bias, strengthening the validity of the results.

Several limitations should be considered. First, the observational design cannot establish causality; residual confounding (e.g., unmeasured dietary factors or medication use affecting electrolyte levels) may influence results [35]. Second, electrolyte measurements were likely single‐point in the third trimester, potentially missing dynamic changes during pregnancy or postpartum. Third, while we adjusted for multiple covariates, unmeasured variables (e.g., genetic polymorphisms) could contribute to residual bias [36]. Finally, the generalizability of findings may be limited to the study population (Chinese women), emphasizing the need for replication in diverse cohorts.

Future research should focus on (1) prospective interventional studies to test whether modifying the Ca/K ratio (e.g., via dietary interventions) reduces postpartum VTE risk; (2) mechanistic studies to elucidate how electrolyte ratios regulate Hb and D‐dimer pathways; (3) longitudinal assessments of electrolyte dynamics during pregnancy to capture temporal associations with VTE; and (4) validation of findings in multi‐ethnic cohorts to confirm global relevance.

In conclusion, this study identifies a serum Ca/K ratio below 0.55 as a novel, independent risk biomarker for postpartum VTE. The association is partially mediated through pathways involving Hb and D‐dimer, suggesting that the risk is linked to anemia‐related pathophysiology. Therefore, our findings advocate for a dual‐pronged preventive approach: optimizing perinatal electrolyte homeostasis alongside vigilant monitoring and management of anemia to mitigate the burden of postpartum VTE.

## 4 Materials and Methods

### 4.1 Data Source

This retrospective multicenter cohort study included pregnant women who attended and delivered at Union Hospital, Tongji Medical College, Huazhong University of Science and Technology, Wuhan, China. Participants were recruited from its three major campuses (the Main Campus, Chegu Campus, and Cancer Center) between January 2017 and December 2024. This study excluded participants with missing electrolyte measurements in the third trimester and those with a history of VTE.

In this study, the sociodemographic information, fertility history, pre‐existing disease history, drug use history, etc., of the participants were collected at the time of inclusion in the third trimester, height and weight were measured at the time of inclusion, and childbirth information was collected during delivery. At 6 weeks postpartum, the onset of postpartum VTE was collected. The diagnosis of postpartum VTE is based on the diagnostic criteria of International Classification of Diseases, 10th Revision (ICD‐10), coding system, and has been verified by multiple researchers and described in detail in our previous studies [37, 38].

All blood samples were tested at enrollment in the third trimester. The serum electrolyte level was measured by I2000 fully automated chemiluminescence immunoassay analyzer (Abbott) and the manufacturer's reagents, with results expressed in mmol/L. Blood routine indicators (including hemoglobin (Hb)) were detected with the Beckman DXH800 Hematology Analyzer. Coagulation indicators were detected by STA‐R Evolution hemagglutination analyzer (Stago, France) and supporting calibrators, quality controls, and reagents. In this study, the exposure variables include single and mixed exposures to six electrolytes (total Ca, Mg, Na, K, P, Cl), the Ca/K ratio (with cut‐off values of 0.553 and 0.55), and different tertile groups of Cl and Mg. To account for physiological hemodilution and its effect on total Ca, albumin‐corrected Ca was calculated for all participants using the following standard formula: albumin‐corrected Ca (mg/dL) = serum total Ca (mg/dL) + 0.8 × [4.0 − serum albumin (g/dL)] [39].

This study followed the Strengthening the Reporting of Observational Studies in Epidemiology (STROBE) reporting guideline for cohort studies.

This study was approved by the ethics committee of Tongji Medical College affiliated with Huazhong University of Science and Technology (number: [2015] S014). This study was a retrospective analysis based on existing medical records. Formal informed consent was waived by the ethics committee due to the anonymous nature of the extracted data and the non‐interventional study design.

### 4.2 Assessment of Covariates

In the present study, covariates were included in the statistical models to adjust for potential confounding effects. Chronic kidney disease indicated a pre‐pregnancy diagnosis of chronic kidney disease (stages 1–5) based on medical records. Autoimmune diseases included pre‐existing autoimmune conditions (e.g., antiphospholipid syndrome) confirmed by medical records.

### 4.3 Statistical Analysis

In this study, categorical data (for the total population, non‐VTE group, and VTE group) were presented as counts (N) and percentages, while continuous data are expressed as median with interquartile range (IQR). Differences in categorical data between groups were compared using the chi‐square test, and differences in continuous data were analyzed using the Mann–Whitney *U* test.

Logistic regression was used to evaluate the association between the Ca/K ratio (with cutoff values of 0.553 and 0.55) and the risk of postpartum VTE. The model was adjusted for the following covariates: maternal age, multiple pregnancy, primiparity, in vitro fertilization pregnancy, ethnicity, baseline body mass index (BMI), smoking status, drinking status, gestational diabetes mellitus (GDM), preeclampsia, preterm birth, delivery mode, postpartum hemorrhage, chronic kidney disease, autoimmune diseases, and serum levels of Mg, Na, P, and Cl. Stratified analyses by delivery mode (cesarean section vs. vaginal delivery) and parity (primiparous vs. multiparous) were performed to further explore the association between the Ca/K ratio and postpartum VTE risk. Sensitivity analyses were conducted to assess the stability of this association by excluding participants with chronic kidney disease, autoimmune diseases, and those enrolled between 2019 and 2020.

Mediation analyses were performed to determine whether Hb, coagulation parameters (e.g., D‐dimer, TT: thrombin time, APTT: activated partial thromboplastin time, PT: prothrombin time, FIB: fibrinogen), and C‐reactive protein (CRP) act as mediating variables in the relationship between the Ca/K ratio and postpartum VTE risk. The selection of these candidate mediators was based on their established pathophysiological links to both electrolyte homeostasis and thrombosis: (1) Hb is associated with blood viscosity and endothelial function, which are key factors in VTE pathogenesis [40], (2) coagulation parameters (D‐dimer, TT, APTT, PT, FIB) directly reflect coagulation status, which is closely related to thrombotic events [41], (3) CRP is a marker of systemic inflammation, and inflammation is an important driver of VTE [42].

Logistic regression was also used to investigate the association between groups defined by low‐Cl+medium‐Mg levels and postpartum VTE risk. This model was adjusted for covariates including maternal age, multiple pregnancy, primiparity, in vitro fertilization pregnancy, ethnicity, baseline BMI, smoking status, drinking status, GDM, preeclampsia, preterm birth, delivery mode, postpartum hemorrhage, chronic kidney disease, autoimmune diseases, and serum levels of Ca, K, Na, and P. Further analyses were conducted to explore whether GDM modifies the effect of low‐Cl+medium‐Mg on postpartum VTE risk.

RCS models (with three knots at the 10th, 50th, and 90th percentiles) were used to analyze the association between six serum electrolytes (Ca, Mg, Na, K, P, Cl, after natural log transformation), Ca/K ratio (albumin‐corrected Ca/K ratio) and postpartum VTE risk, with values outside the fifth and 95th percentiles excluded.

Weighted quantile sum (WQS) regression was applied to evaluate the combined contribution of the six electrolytes to postpartum VTE risk. Bayesian kernel machine regression (BKMR) was used to assess the joint effect of six electrolytes on the risk of postpartum VTE, and the impact of an individual electrolyte as part of an electrolyte mixture. We used a probit link function with BKMR in consideration of present binary outcome (VTE or non‐VTE) [43]. BKMR models estimated the PIP of each electrolyte in relation to postpartum VTE risk and to assess the combined effects of mixed exposures to the six electrolytes.

Serum Cl and Mg levels were divided into tertiles. Logistic regression was used to compare the risk of postpartum VTE across different Cl and Mg tertile groups, with the medium‐Cl and low‐Mg group as the reference. The correlation between the six electrolytes was analyzed by Spearman correlation analysis.

Data analyses were performed using R. A two‐sided *p* value <0.05 was considered statistically significant.

## Author Contributions

**Qian Li**: conceptualization (lead), writing – original draft (lead), formal analysis (lead), writing – review and editing (lead), funding acquisition. **Guofu Zhang**: data curation, methodology, software, writing – review and editing. **Xiating Li**: data curation, writing – review and editing. **Huafang Wang**: data curation, writing – review and editing. **Jun Deng**: data curation, writing – review and editing. **Zhipeng Cheng**: data curation, writing – review and editing, funding acquisition. **Fengjuan Fan**: data curation, writing – review and editing. **Shi Chen**: data curation, writing – review and editing. **De Li**: data curation, writing – review and editing. **Liang V. Tang**: conceptualization, project administration (lead), writing – review and editing, funding acquisition. **Yu Hu**: conceptualization, project administration (lead), writing – review and editing, funding acquisition. All authors have read and approved the final manuscript.

## Funding

This study is supported by the National Natural Science Foundation of China (Nos. 82103827 and 82170131), the Young Elite Scientists Sponsorship Program by CAST (2023QNRC001), and Program of National Key Research and Development Project of China (Nos. 2022YFC2304600 and 2023YFC2509500).

## Ethics Statement

This study was approved by the ethics committee of Tongji Medical College affiliated with Huazhong University of Science and Technology (no: [2015] S014). Informed consent was not required since the information was retrieved through the medical records retrospectively.

## Conflicts of Interest

All authors declare no conflicts of interest.

## Supporting information




**Supporting file 1**: mco270843‐sup‐0001‐SuppMat.pdf

## Data Availability

All data generated or analyzed during this study are included in this published article and its Supporting Information.
