# Supplementary material for: A Low Serum Calcium‐to‐Potassium Ratio Predicts Postpartum Venous Thromboembolism Risk: Mediation by Hemoglobin and D‐Dimer in a Multicenter Cohort
Source: MedComm (2020). 2026 Jun 25;7(7):e70843. doi: 10.1002/mco2.70843 (PMC13296241; doi:10.1002/mco2.70843)
Supplement: Supplementary file 1 — Supporting file 1: mco270843‐sup‐0001‐SuppMat.pdf [file MCO2-7-e70843-s001.pdf]

Title: A low serum calcium-to-potassium ratio predicts postpartum venous thromboembolism risk: mediation by hemoglobin and D-dimer in a multicenter cohort

A running title: Calcium-potassium ratio and postpartum VTE

Authors: Qian Li[1,2], PhD, Guofu Zhang[3], PhD, Xiating Li[4], MPH, Huafang Wang[1,2], PhD, Jun Deng[1,2], PhD, Zhipeng Cheng[1,2], PhD, Fengjuan Fan[1,2], PhD, Shi Chen[5], MPH, De Li[5], MPH, Liang V Tang[1,2,6],* PhD, Yu Hu[1,2,6], *PhD

*Contributed equally

Affiliations:

[1]Institute of Haematology, Union Hospital, Tongji Medical College, Huazhong University of Science and Technology, Wuhan, Hubei, China

[2]Key Laboratory of Biological Targeted Therapy (Huazhong University of Science and Technology), Ministry of Education, Wuhan, Hubei, China

[3]School of Public Health, Xinxiang Medical University, Xinxiang, Henan, China

[4]Diagnostic Science Centre, Alberta Precision Laboratories, Calgary, AB, Canada

[5]Department of Biobank, Union Hospital, Tongji Medical College, Huazhong University of Science and Technology, Wuhan, Hubei, China

[6]Collaborative Innovation Center of Hematology, Huazhong University of Science and Technology, Wuhan, Hubei, China

Correspondence to:

Yu Hu, Email: huyu_whuh@hust.edu.cn

Liang V Tang, Email: lancet.tang@qq.com

Table S1—Characteristics of the participants included and excluded from this study.

	Total (n = 32241)	Included (n=25792)	Excluded (n=6449)
**Characteristics**			
**Maternal age (years)**	31.0 (28.0, 34.0)	31.0 (28.0, 34.0)	30.0 (27.0, 33.0)
**Ethnic group (Han)**	32047 (99.4)	25622 (99.3)	6425 (99.6)
**Multiple pregnancy (%)**	1198 (3.7)	1094 (4.2)	104 (1.6)
**Primipara (%)**	19956 (61.9)	16807 (65.2)	3149 (48.8)
**IVF pregnancy (%)**	2279 (7.1)	2035 (7.9)	244 (3.8)
**Height at enrollment (cm)**	161.0 (158.0, 165.0)	161.0 (158.0, 165.0)	160.0 (158.0, 164.0)
**Weight at enrollment (kg)**	69.0 (63.0, 75.0)	69.0 (63.0, 75.0)	69.0 (63.0, 75.0)
**Habit of smoking (%)**	48 (0.1)	38 (0.1)	10 (0.2)
**Habit of drinking (%)**	33 (0.1)	24 (0.1)	9 (0.1)
**GDM (%)**	7709 (23.9)	5879 (22.8)	1830 (28.4)
**Preeclampsia (%)**	1311 (4.1)	1193 (4.6)	118 (1.8)

Abbreviations: GDM: gestational diabetes mellitus.

Table S2—Characteristics of the participants included in different year.

	Included in 2017-2024 (n=25792)	Included in 2017, 2018, 2021-2024 (n=22985)	Included in 2019-2020 (n=2807)
**Characteristics**			
**Maternal age (years)**	31.0 (28.0, 34.0)	31.0 (28.0, 34.0)	30.0 (28.0, 34.0)
**Ethnic group (Han)**	25622 (99.3)	22821 (99.3)	2801 (99.8)
**Multiple pregnancy (%)**	1094 (4.2)	922 (4.0)	172 (6.1)
**Primipara (%)**	16807 (65.2)	15098 (65.7)	1709 (60.9)
**IVF pregnancy (%)**	2035 (7.9)	1791 (7.8)	244 (8.7)
**Height at enrollment (cm)**	161.0 (158.0, 165.0)	161.0 (158.0, 165.0)	161.0 (158.0, 165.0)
**Weight at enrollment (kg)**	69.0 (63.0, 75.0)	69.0 (63.0, 75.0)	69.0 (63.0, 75.0)
**Habit of smoking (%)**	38 (0.1)	30 (0.1)	8 (0.3)
**Habit of drinking (%)**	24 (0.1)	20 (0.1)	4 (0.1)
**GDM (%)**	5879 (22.8)	5283 (23.0)	596 (21.2)
**Preeclampsia (%)**	1193 (4.6)	1018 (4.4)	175 (6.2)

Abbreviations: GDM: gestational diabetes mellitus.

Table S3—Sensitivity analysis of calcium-potassium (Ca/K) ratio and postpartum VTE risk.

Categories	Ca/K ratio	
	**<0.55**	**≥0.55**
**Excluding participants with chronic kidney disease (n=25704)**	OR (95%CI)[a] 1.66 (1.10, 2.49)*	Ref.
**Excluding participants with autoimmune diseases (n=25631)**	OR (95%CI)[b] 1.70 (1.13, 2.56)*	Ref.
**Excluding participants enrolled in 2019-2020 (n=22985)**	OR (95%CI)[c] 1.69 (1.08, 2.63)*	Ref.

Abbreviations: Ca: calcium; K: potassium; OR: odds ratio; VTE: venous thromboembolism.

*$P< 0.05$

[a]Adjusted for maternal age, multiple pregnancy, primipara, *in vitro* fertilisation pregnancy, ethnic group, BMI at enrollment, habit of smoking, habit of drinking, gestational diabetes mellitus, preeclampsia, preterm, delivery mode, postpartum haemorrhage, autoimmune diseases, serum magnesium, serum sodium, serum phosphorus and serum chlorine.

[b]Adjusted for maternal age, multiple pregnancy, primipara, *in vitro* fertilisation pregnancy, ethnic group, BMI at enrollment, habit of smoking, habit of drinking, gestational diabetes mellitus, preeclampsia, preterm, delivery mode, postpartum haemorrhage, chronic kidney disease, serum magnesium, serum sodium, serum phosphorus and serum chlorine.

[c]Adjusted for maternal age, multiple pregnancy, primipara, *in vitro* fertilisation pregnancy, ethnic group, BMI at enrollment, habit of smoking, habit of drinking, gestational diabetes mellitus, preeclampsia, preterm, delivery mode, postpartum haemorrhage, chronic kidney disease, autoimmune diseases, serum magnesium, serum sodium, serum phosphorus and serum chlorine.

Table S4—Mediated effects on associations between calcium-potassium (Ca/K) ratio and postpartum VTE risk.

Potential mediating variables	Total effect (OR, 95% CI)	Mediating effect (OR, 95% CI)	Mediating proportion (%)	P-value (mediating effect)
**Hb**	0.91 (0.67, 0.99)	0.97 (0.91, 0.99)	34.7	0.036*
**D-dimer**	0.86 (0.61, 0.99)	0.99 (0.98, 1.00)	4.6	0.018*
**TT**	0.87 (0.62, 0.99)	1.03 (0.99, 1.08)	-	-
**APTT**	0.85 (0.60, 0.99)	1.01 (0.99, 1.03)	-	-
**PT**	0.87 (0.62, 0.99)	1.00 (0.99, 1.01)	-	-
**Fib**	0.88 (0.64, 0.99)	0.99 (0.99, 1.00)	-	-
**CRP**	0.78 (0.52, 0.99)	1.00 (0.99, 1.00)	-	-

Abbreviations: APTT: activated partial thromboplastin time; Ca: calcium; CRP: C-reactive protein; Fib: fibrinogen; K: potassium; OR: odds ratio; PT: prothrombin time; TT: thrombin time; VTE: venous thromboembolism.

*$P < 0.05$

Adjusted for maternal age, multiple pregnancy, primipara, *in vitro* fertilisation pregnancy, ethnic group, BMI at enrollment, habit of smoking, habit of drinking, gestational diabetes mellitus, preeclampsia, preterm, delivery mode, postpartum haemorrhage, chronic kidney disease, autoimmune diseases, serum magnesium, serum sodium, serum phosphorus and serum chlorine.

Table S5—Total and stratified analyses of the association between low-chlorine + medium-magnesium group (vs. other groups) and postpartum VTE risk by GDM (n=25792).

Low-Cl+medium-Mg	Unadjusted	Adjusted
Yes	1.83 (1.18, 2.85)*	2.11 (1.34, 3.31)[a]*
No	Ref.	Ref.

Low-Cl+medium-Mg		
**Stratified by GDM**		
GDM	1.91 (0.83, 4.39)	2.27 (0.97, 5.29)
Non-GDM	1.83 (1.08, 3.08)*	2.04 (1.20, 3.48)[b]*

Abbreviations: APTT: activated partial thromboplastin time; Cl: chlorine; CRP: C-reactive protein; Fib: fibrinogen; K: potassium; Mg: magnesium; OR: odds ratio; PT: prothrombin time; TT: thrombin time; VTE: venous thromboembolism.

*$P< 0.05$

[a]Adjusted for maternal age, multiple pregnancy, primipara, *in vitro* fertilisation pregnancy, ethnic group, BMI at enrollment, habit of smoking, habit of drinking, gestational diabetes mellitus, preeclampsia, preterm, delivery mode, postpartum haemorrhage, chronic kidney disease, autoimmune diseases, serum calcium, serum potassium, serum sodium, and serum phosphorus.

[b]Adjusted for maternal age, multiple pregnancy, primipara, *in vitro* fertilisation pregnancy, ethnic group, BMI at enrollment, habit of smoking, habit of drinking, preeclampsia, preterm, delivery mode, postpartum haemorrhage, chronic kidney disease, autoimmune diseases, serum calcium, serum potassium, serum sodium, and serum phosphorus.

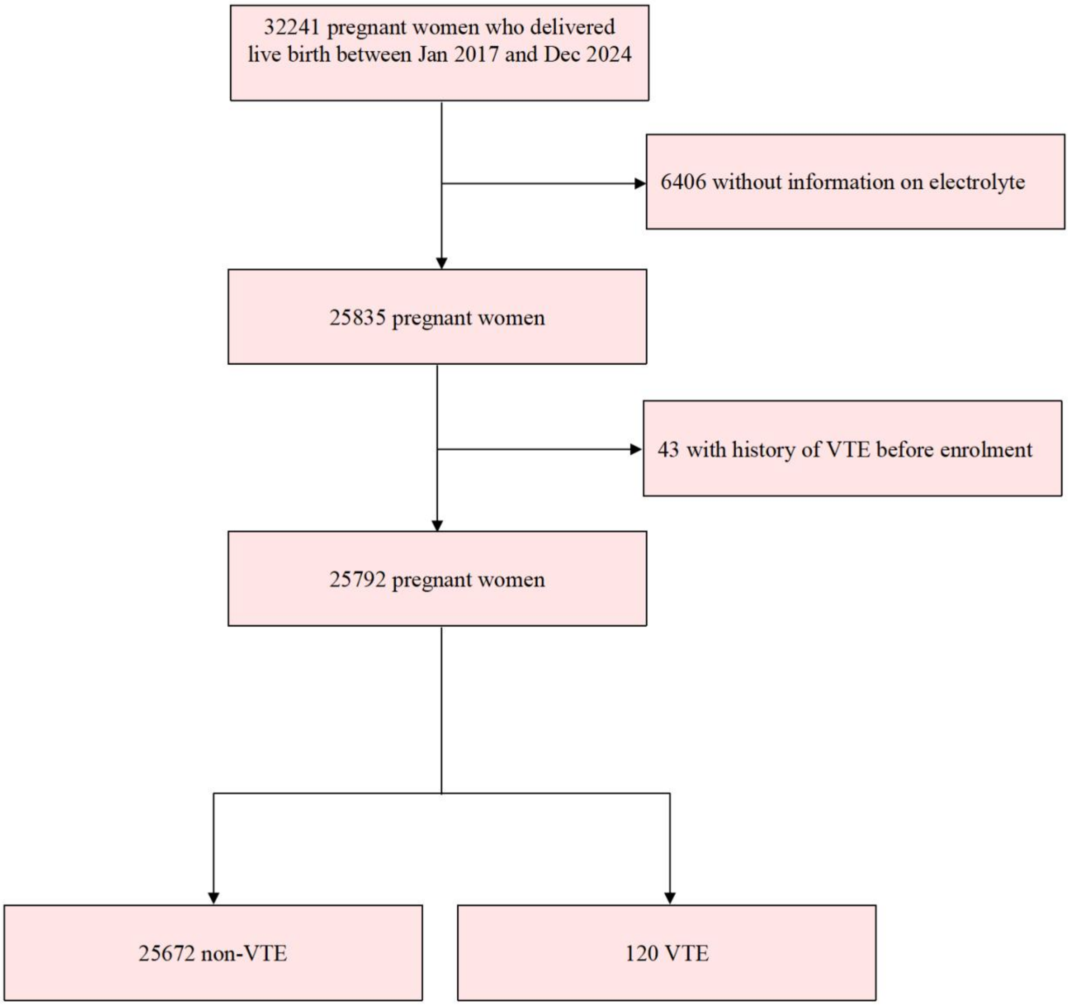

Figure S1——Flow diagram of this study.

Abbreviations: VTE: venous thromboembolism

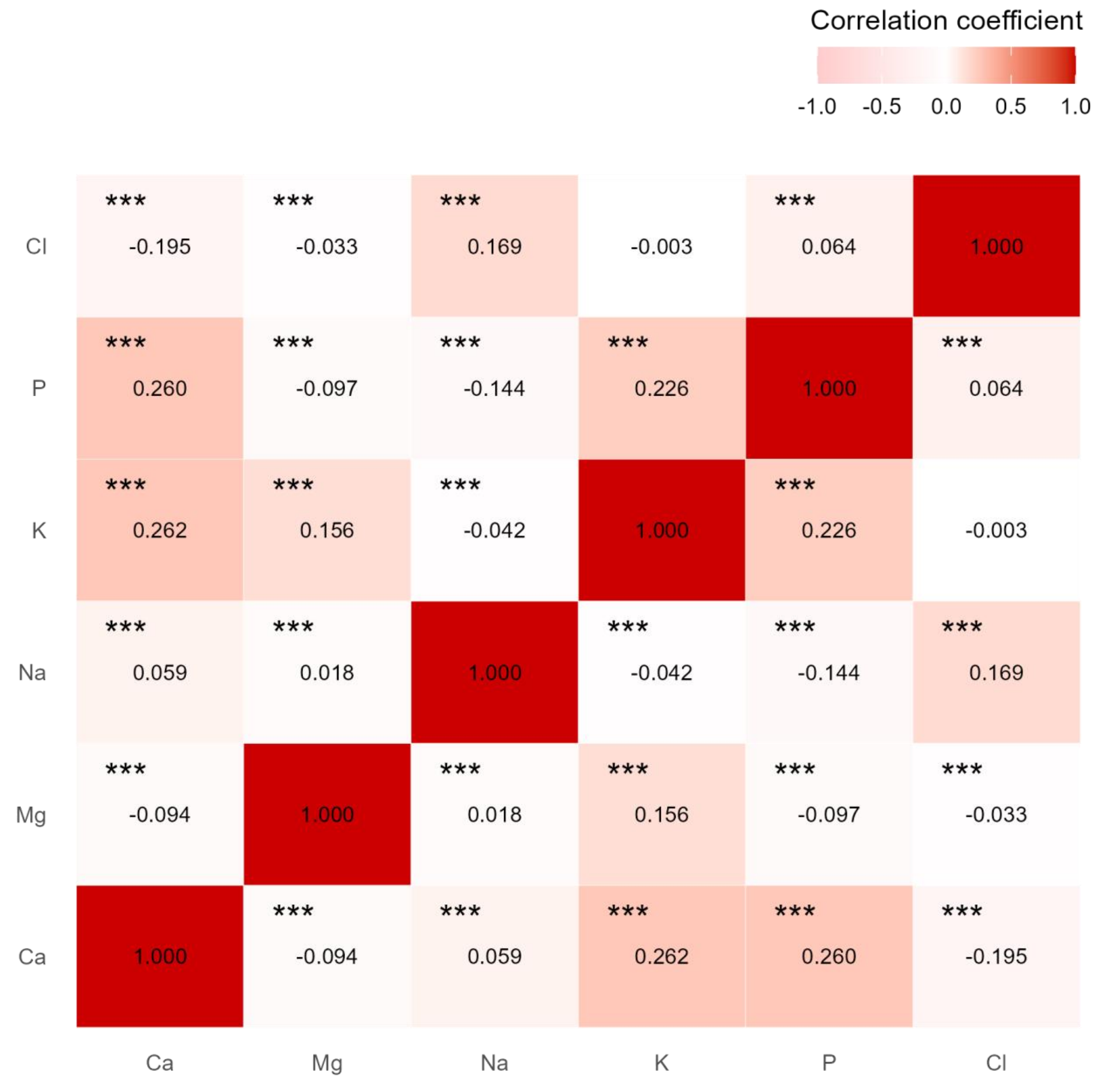

Figure S2——Correlation analysis of six serum electrolytes (n=25792).

Abbreviations: Ca: calcium; Cl: chlorine; K: potassium; Mg: magnesium; Na: sodium; P: phosphorus.

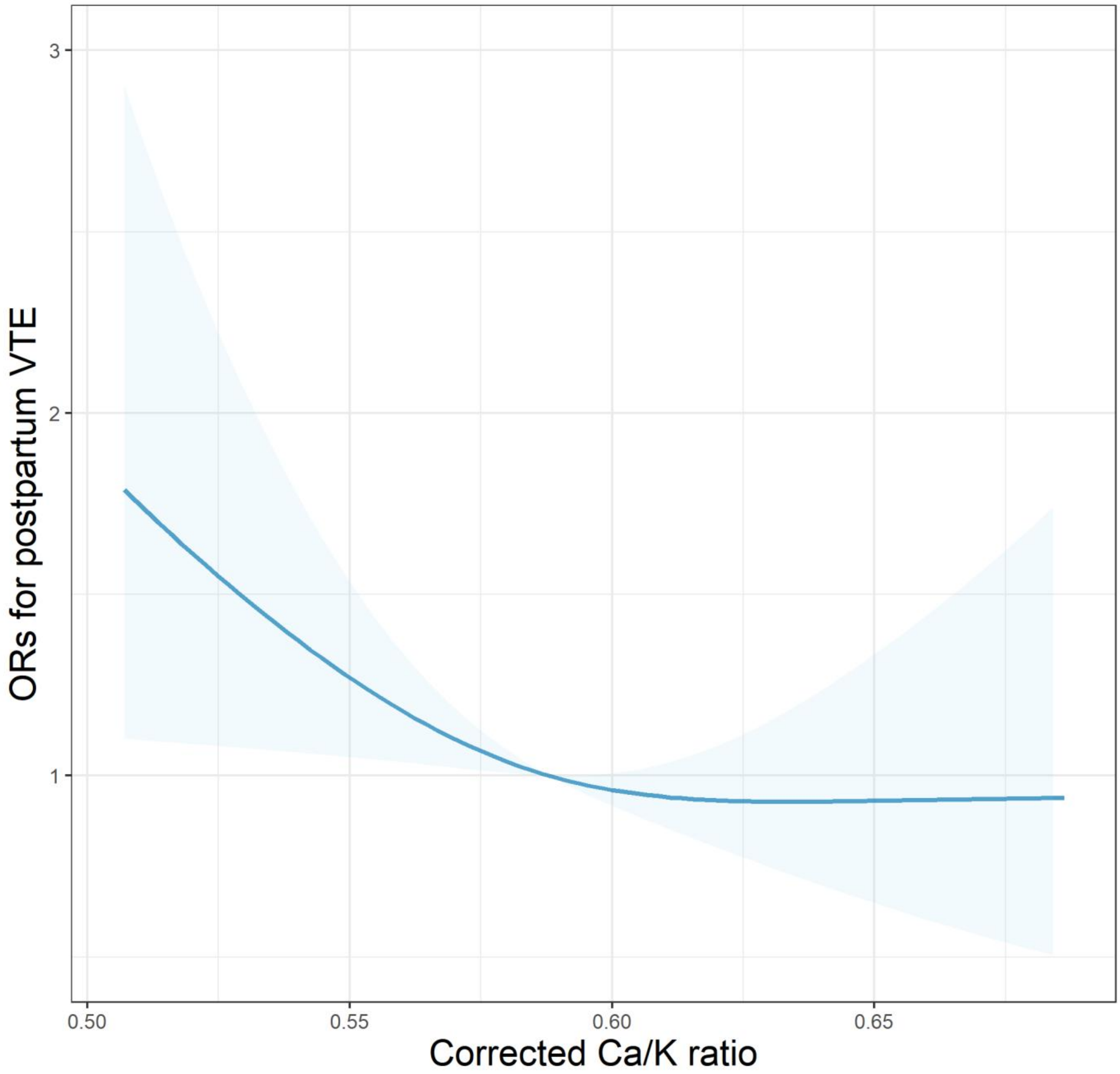

Figure S3——Restricted Cubic Spline (RCS) analyses of associations between albumin-corrected Ca/K ratio and postpartum VTE.

Abbreviations: Ca: calcium; K: potassium; OR: odds ratio; VTE: venous thromboembolism.

Models adjusted for maternal age, multiple pregnancy, primipara, *in vitro* fertilisation pregnancy, ethnic group, BMI at enrollment, habit of smoking, habit of drinking, gestational diabetes mellitus, preeclampsia, preterm, delivery mode, postpartum haemorrhage, chronic kidney disease, autoimmune diseases, serum magnesium, serum sodium, serum phosphorus and serum chlorine.

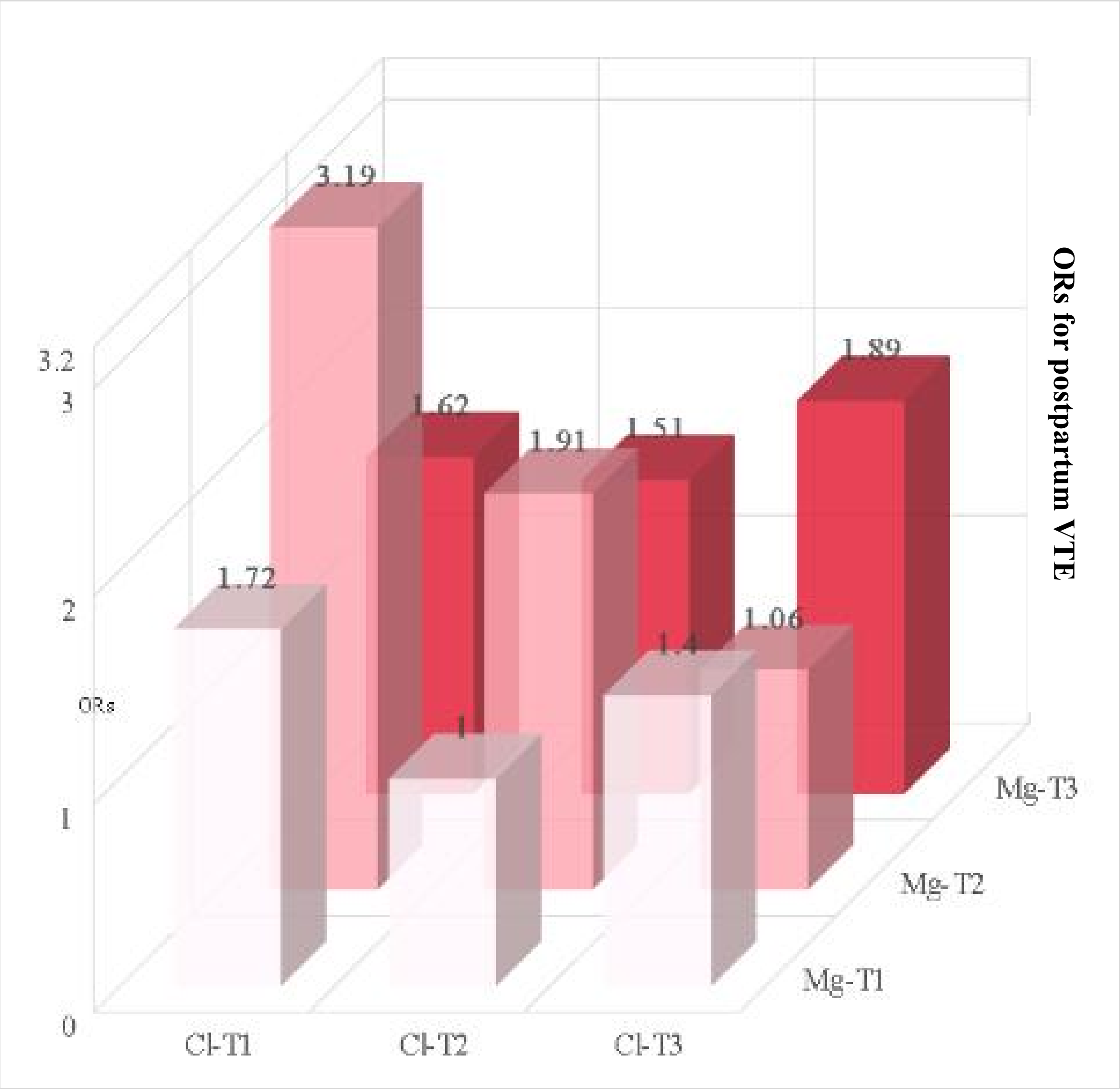

Figure S4——Postpartum VTE risk differences across chloride (Cl) and magnesium (Mg) tertiles.

Abbreviations: Cl: chlorine; Mg: magnesium; OR: odds ratio; VTE: venous thromboembolism.

Adjusted for maternal age, multiple pregnancy, primipara, *in vitro* fertilisation pregnancy, ethnic group, BMI at enrollment, habit of smoking, habit of drinking, gestational diabetes mellitus, preeclampsia, preterm, delivery mode, postpartum haemorrhage, chronic kidney disease, autoimmune diseases, serum calcium, serum potassium, serum sodium, and serum phosphorus.
